# From Yellow Cumulus to Pregnancy in Autoimmune Hemolytic Anemia Following Bilirubin Reduction: A Case Report

**DOI:** 10.7759/cureus.88363

**Published:** 2025-07-20

**Authors:** Feras Sendy, Simon Phillips, Sylvain Ménard

**Affiliations:** 1 Obstetrics and Gynecology, King Fahad Medical City, Riyadh, SAU; 2 Reproductive Endocrinology and Infertility, University of Montreal Health Centre (CHUM), Montreal, CAN; 3 Reproductive Endocrinology and Infertility, Ovo Fertility Clinic, Montreal, CAN; 4 Obstetrics and Gynecology, University of Montreal Health Centre (CHUM), Montreal, CAN

**Keywords:** autoimmune hemolytic anemia, bilirubin levels, embryo transfer, in vitro fertilization, pregnancy

## Abstract

Autoimmune hemolytic anemia (AIHA) is a systemic condition associated with inflammation and oxidative stress, both of which can negatively affect reproductive outcomes. Bilirubin, a byproduct of hemolysis, has been implicated in oocyte maturation and embryogenesis. We present the case of a 31-year-old patient with primary infertility who was known with AIHA. She had a total bilirubin level of 60 µmol/L prior to the first stimulation cycle, which was associated with yellowish pigmentation of the cumulus cells, poor oocyte quality, and no blastocyst formation. The decline in the total bilirubin level to 48 µmol/L after prednisone therapy of 60 mg daily for three months led to improved cumulus cell appearance and enhanced oocyte and embryo quality in the second stimulation cycle. A successful pregnancy was achieved following frozen blastocyst transfer in a natural cycle. This case underscores the importance of individualized assessment and the careful interpretation of elevated bilirubin levels in the context of assisted reproductive technology, as systemic conditions may potentially impact reproductive success.

## Introduction

Age is a known pillar that impacts oocyte quality, fertilization, and embryo development leading to pregnancy [1]. The oocyte floats in the follicular fluid (FF), which serves as a reservoir for various substances produced by granulosa and theca cells [2,3]. Bilirubin is a yellow pigment (a breakdown product of heme metabolism) that has been reported in the FF [2,3]. Oocyte maturation and fertilization leading to embryo development are linked to bilirubin as it could be a potential marker of oxidative stress within the ovarian follicle [4,5]. Autoimmune hemolytic anemia (AIHA), reduced ovarian reserve (ROR), endometriosis, and polycystic ovarian syndrome (PCOS) are associated with oxidative stress and inflammation through different pathophysiological mechanisms, which can adversely affect reproductive outcomes [6-9]. Patients with AIHA are characterized by destruction of the red blood cells, leading to elevated bilirubin levels, promoting oxidative damage, which can impair folliculogenesis and embryo quality [4,5,9].

## Case presentation

A 31-year-old nulliparous woman presented to Ovo Fertility Center with a one-year history of primary infertility due to combined factors including low ovarian reserve and oligospermia. Her medical history was remarkable for AIHA and a known penicillin allergy. She had a body mass index (BMI) of 21 kg/m^2^, a regular menstrual cycle, and a contradictory ovarian reserve with an anti-Müllerian hormone (AMH) level of 1.54 ng/mL (reference range: 2-4 ng/mL) and an antral follicle count (AFC) of 29 (reference range: 6-10 by ovary). Her partner, a 30-year-old male, was diagnosed with severe oligospermia. Semen analysis revealed a sperm concentration of 1.08 million/mL, progressive motility of 48%, and normal morphology of 11%. His DNA fragmentation index was at 2.6%. Genetic testing for Y-chromosome microdeletions and karyotyping yielded normal results. Intracytoplasmic sperm injection (ICSI) was recommended.

The patient underwent ovarian stimulation using an antagonist protocol (daily Gonal-F^®^ 225 IU and Luveris^®^ 75 IU) and Aspirin^®^ 81 mg daily to reduce the risk of thrombosis. The doses of medications were increased on the sixth day of stimulation (Gonal-F^®^ 450 IU and Luveris^®^ 225 IU) due to inadequate response. Cetrorelix (Cetrotide^®^ 250 mcg) was used for suppression and a trigger by Suprefact^®^ 1 mg alone due to the risk of ovarian hyperstimulation. The aspirated FF was yellow. Fourteen oocytes were retrieved with a yellowish appearance of the cumulus cells, of which seven were mature and used for ICSI. Five oocytes were fertilized, yet none developed into blastocysts.

A multidisciplinary team (fertility specialists, hematologists, and embryologists) recommended the use of Prednisone^®^ 60 mg to reduce the total bilirubin level, which was at 60 µmol/L (reference range: 7-34 µmol/L) due to its potential impact on hemolysis, oocytes, and embryo quality. The patient took Prednisone^®^ 60 mg for two months daily prior to fertility treatment, followed by another month (synchronization prior to ovarian stimulation with Estrace^®^ 4 mg daily from day 20 followed by starting ovarian stimulation medications according to the menstrual cycle), leading to a decrease in total bilirubin to 48 µmol/L.

The patient underwent a second ovarian stimulation using an antagonist protocol (daily Menopur^®^ 300 IU and Rekovelle^®^ 12 mcg), Aspirin^®^ 81 mg, and Prednisone^®^ 60 mg daily. Cetrorelix (Cetrotide^®^ 250 mcg) was used for suppression and a dual trigger (human chorionic gonadotropin^®^ (hCG^®^) 5,000 IU and Suprefact^®^ 1 mg). On the trigger day, nine follicles ≥ 14 mm were detected, with estradiol and progesterone levels of 8,206 pmol/L (reference range: 917.8-1,101.4 pmol/L by mature follicle) and 2.66 nmol/L (reference range: <4.7 nmol/L to avoid premature luteinization), respectively. The aspirated FF was yellow in color, as in previous oocyte collection, yet there was no yellowish appearance of the cumulus cells under the microscope. Seventeen oocytes were retrieved, of which five were mature and used for ICSI. Four oocytes were fertilized, and two blastocysts were obtained and vitrified (a day 5 Grade 3AA and a day 6 Grade 2AB). The visual grading of embryos under the microscope by the embryologist is based on blastocyst expansion, quality of the trophectoderm, and inner cell mass. Prednisone^®^ 60 mg was tapered off 10 mg every week after the oocyte retrieval, leading to complete withdrawal five weeks prior to frozen embryo transfer.

A single Grade 3AA day 5 blastocyst was transferred on a frozen natural cycle. Luteal phase support was done with vaginal progesterone (Crinone^®^ 8% twice daily). Her first hCG 10 days post transfer was 351 mIU/mL, followed by 815 mIU/mL 48 hours later. Therefore, she was advised to continue progesterone up to 10 weeks of gestation. The transvaginal ultrasound revealed an intrauterine gestational sac with a positive yolk sac, fetal pole, and a positive fetal cardiac activity, with a crown rump length (CRL) corresponding to seven weeks and four days (seven weeks and three days according to the day of embryo transfer). All her hCG blood tests were done in the same laboratory. Patient consent was obtained for personal medical information and the publication of this case report.

## Discussion

This is the first reported case of a successful pregnancy after frozen blastocyst transfer in a natural cycle after a second ovarian stimulation cycle with prednisone therapy. Prednisone was suggested after a multidisciplinary team discussion regarding the patient’s medical condition with AIHA, bilirubin levels prior to the stimulation, ROR, response to the first stimulation cycle, yellow pigmentation of the FF and cumulus cells, maturation, fertilization rates, and the absence of blastocysts to transfer. The objective of the treatment with prednisone was to reduce hemolysis, leading to a decrease in bilirubin levels and oxidative stress, ultimately resulting in a better response to ovarian stimulation, reduction, or removal of the yellowish coloration of the FF and cumulus cells, and improving maturation, fertilization, and blastocyst rates in our patient. A comparison between the first and second stimulation cycles is summarized in Table 1.

**Table 1:** Summary of the first and second stimulation cycles (medications, days of stimulation, progesterone and estradiol levels during the ovarian stimulation, ovulation induction medication, number of follicles ≥ 14 mm on the day of ovulation induction, total number of oocytes, number of mature oocytes, bilirubin levels, follicular fluid color, cumulus cell color, oocyte quality, number of a germinal vesicle (GV), metaphase 1 (M1), degenerated, empty zona (EZ), maturation, fertilization, and blastocyst rates. *It is unknown if some of the degenerated oocytes could have been mature, which may impact the maturation rate. Total number of oocytes: number of mature and immature oocytes; oocyte quality: morphological criteria: cytoplasmic morphology including organelles, perivitelline space, and zona pellucida to determine oocyte quality (good or poor); maturation rate: number of mature oocytes divided by the number of follicles ≥ 14 mm; fertilization rate: number of pronuclei (2PN) divided by the number of mature oocytes; blastocyst rate: number of blastocysts divided by the number of 2PN. Suprefact^®^ is the brand name for buserelin, a gonadotropin-releasing hormone (GnRH) agonist used for ovulation induction. hCG^®^ is a human chorionic gonadotropin used for ovulation induction. Gonal-F^®^ is the brand name for follitropin alfa, a recombinant form of follicle-stimulating hormone (FSH). Luveris^®^ is the brand name for lutropin alfa, a recombinant form of luteinizing hormone (LH). Menopur^®^ is the brand name for menotropins, a combination of FSH and LH. Rekovelle^®^ is the brand name for follitropin delta, a recombinant FSH. Aspirin^®^ is the brand name for acetylsalicylic acid used to prevent blood clots. Prednisone^®^ is a drug used to suppress the immune system and reduce inflammation. Cetrotide^®^ is the brand name for cetrorelix acetate, a GnRH antagonist used to prevent premature ovulation.

	1st cycle	2nd cycle
Medication	Aspirin^®^ 81 mg	Aspirin^®^ 81 mg
	Gonal-F^®^ 225 IU	Menopur^®^ 300 IU
	Luveris^®^ 75 IU	Rekovelle^®^ 12 mcg
	Cetrotide^®^ 250 mcg	Cetrotide^®^ 250 mcg
	-	Prednisone^®^ 60 mg
Days of stimulation	14 days	13 days
Estradiol on day 6	588 pmol/L	675 pmol/L
Estradiol (E2) the day of ovulation induction	-	E2: 8,206 pmol/L
Progesterone (P4) the day of ovulation induction	-	P4: 2.66 nmol/L
E2 one day prior to ovulation induction	E2: 10,003 pmol/L	-
P4 one day prior to ovulation induction	P4: 2.71 nmol/L	-
Ovulation induction medication	Suprefact^®^ 1 mg	Suprefact^®^ 1 mg
	-	hCG^®^ 5,000 IU
Number of follicles ≥ 14 mm on the day of ovulation induction	8	9
Total number of oocytes	14	17
Number of mature oocytes	7	5
Bilirubin level	60 µmol/L	48 µmol/L
Follicular fluid color	Yellow	Yellow
Cumulus cell color	Yellow	Not yellow
Oocyte quality	Poor quality	Poor quality
Number of germinal vesicle (GV)	7	4
Number of metaphase 1 (M1)	0	2
Number of degenerated^*^	0	5
Number of empty zona (EZ)	0	1
Maturation rate	87.5%	55.5%
Fertilization rate	71.4%	80%
Blastocyst rate	0%	50%

After achieving the objective of reducing bilirubin levels with prednisone, ovarian stimulation and oocyte collection resulted in the collection of cumulus cells without yellow pigmentation and the production of two blastocysts. No prior case in the literature describes such a procedure. This underlines the unique nature of this case and its potential to challenge the existing framework with increased oxidative stress.

AIHA is characterized by hemolysis, and increased bilirubin levels are one of the hallmarks of hemolysis [10]. It has been described that some erythrocytes form microspherocytes, which are characterized by rigid cellular membranes and lack flexibility, leading to hypoxia based on the degree of hemolysis [11,12]. Furthermore, FF in infertile women has been compared to FF in fertile women, and it was found that infertile women were associated with a higher bilirubin level than fertile women [5]. Although the study did not evaluate infertile women with AIHA, they included ROR, which can arguably be linked to AIHA as it is associated with oxidative stress and inflammation. Since bilirubin undergoes changes in the FF of infertile women, it has been proven to have a negative impact on the number of fertilized oocytes, blastocysts, pregnancy, and live birth rates [5]. Such findings are in correlation with our case as oxidative stress to the follicle growth and development plays a role in adverse reproductive outcomes.

The yellowish pigmentation of the FF has been evaluated in a study using spectrophotometric analysis, and it was found that bilirubin is the major contributor to the FF yellow color [4]. The study argues that pigmentation is linked to oocyte maturation, and bilirubin could be a marker of the intraovarian process involved in oocyte maturation. In our case, we did not test the FF for bilirubin levels; however, it is probable that bilirubin would be present as a major marker as proven in the literature. The yellow pigmentation of the FF and cumulus cells in the first stimulation may emphasize the degree of hemolysis and oxidative stress on a cellular level resulting in unfavorable outcomes. The adaptation of treatment with prednisone to reduce hemolysis in the second stimulation cycle led to an improvement in fertilization and blastocyst rates in our case yet with the persistence of pigmentation in the FF.

The strength of our case is that it provided a similar treatment regime with an antagonist protocol, even if the medication for stimulation was slightly different. The same laboratory setup included incubators, culture media, and fertilization techniques. The main difference between the first and second stimulation cycles was the inclusion of prednisone, which improved outcomes in our patient.

The limitation of our case is that we were unable to determine whether the maturation rate was better in the second cycle compared to the first, as some of the degenerated oocytes could have been mature oocytes. Hypothetically, poor oocyte quality could be a reason for degeneration during either the oocyte collection or oocyte stripping (cumulus cell removal) in the laboratory. Another limitation is the absence of bilirubin levels in the FF in both oocyte retrieval cycles. Having bilirubin levels in both the FF and blood tests would have been ideal. However, the application of that was not possible as the multidisciplinary team was after the first oocyte retrieval. Thus, obtaining bilirubin levels in the FF from the first cycle was not feasible. Lastly, further research is needed to confirm our findings, even if we have found better outcomes in our case.

## Conclusions

Reducing bilirubin levels in a patient with AIHA may improve oocyte and embryo quality, ultimately leading to successful in vitro fertilization (IVF) outcomes. The potential impact of systemic conditions on reproductive success, particularly how hemolysis and oxidative stress impact the FF and oocyte development, should be considered. The importance of individualized assessment and careful interpretation of elevated bilirubin levels in the context of assisted reproductive technology (ART) rather than reproductive parameters alone is emphasized. Moreover, the report suggests prednisone therapy may optimize ovarian response and embryo development in selected cases. However, the observations are based on a single case, and further research is needed to validate those findings.
